# Advances in the application of gas vesicles in medical imaging and disease treatment

**DOI:** 10.1186/s13036-024-00426-3

**Published:** 2024-07-23

**Authors:** Renjie Feng, Jie Lan, Meei Chyn Goh, Meng Du, Zhiyi Chen

**Affiliations:** 1 Key Laboratory of Medical Imaging Precision Theranostics and Radiation Protection, College of Hunan Province, the Affiliated Changsha Central Hospital, Hengyang Medical School, University of South China, Changsha, China; 2 Institute of Medical Imaging, Hengyang Medical School, University of South China, Hengyang, China; 3 The Seventh Affiliated Hospital, Hunan Veterans Administration Hospital, Hengyang Medical School, University of South China, Changsha, Hunan China

**Keywords:** Gas vesicles, Microorganism, Imaging, Delivery, Vaccine

## Abstract

The gas vesicle (GV) is like a hollow nanoparticle consisting of an internal gas and a protein shell, which mainly consists of hydrophobic gas vesicle protein A (GvpA) and GvpC attached to the surface. GVs, first discovered in cyanobacteria, are mainly produced by photosynthetic bacteria (PSB) and halophilic archaea. After being modified and engineered, GVs can be utilized as contrast agents, delivery carriers, and immunological boosters for disease prevention, diagnosis, and treatment with good results due to their tiny size, strong stability and non-toxicity advantages. Many diagnostic and therapeutic approaches based on GV are currently under development. In this review, we discuss the source, function, physical and chemical properties of GV, focus on the current application progress of GV, and put forward the possible application prospect and development direction of GV in the future.

## Introduction

Gas vesicles (GVs) are a type of gene-encoded, inert, hollow, gas-filled protein nanoparticles discovered by German microbiologists in cyanobacteria that formed blooms over a century ago [1–3]. Mature GVs are spindle or cylinder organelles that are 0.045–0.2 μm wide and 0.1–2 μm long [4]. GVs are commonly produced by bacteria and archaea, particularly photosynthetic bacteria (PSB) and halophilic archaea. Notably, the GV shell differs from the organelles of other microorganisms in that it is permeable to gases but not to liquid water. It consists only of proteins and does not contain lipids or carbohydrates. The GV shell is only 2 nm thick and consists of a layer of gas vesicle protein A (GvpA), a protein with a molecular weight of 7–8 kDa. This protein forms a 4.6 nm helical stripe perpendicular to the long axis of the vesicle [5]. The physical properties of GV produced by microorganisms similar to PSB and halophilic archaea can provide buoyancy for them to move up and down the water to obtain nutrients [6].

**Fig. 1:**
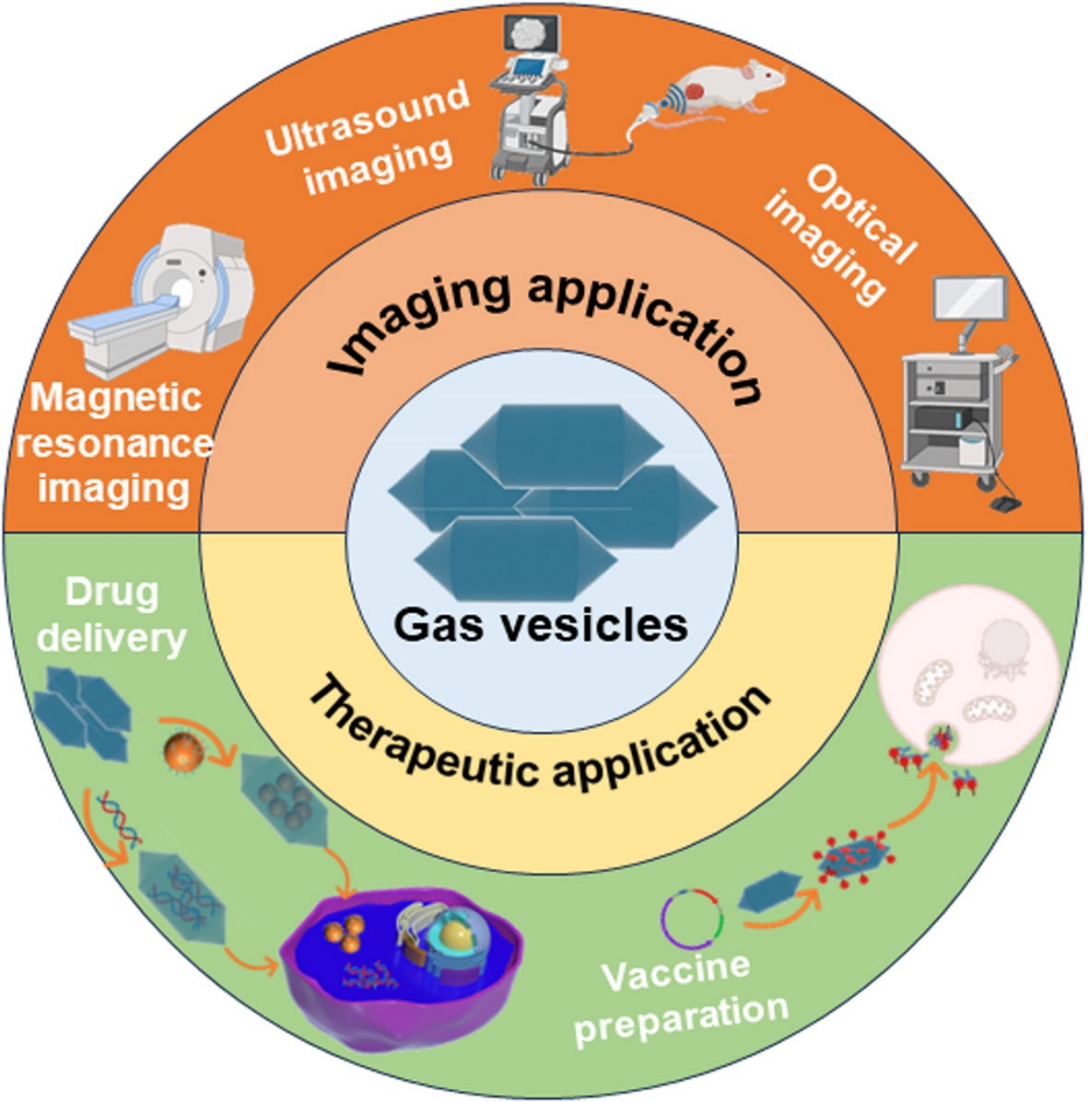
The application of GV in biomedicine

GV, with its distinctive structure similar to the air chamber of a submarine and as a protein assembly, has the potential to be used in a variety of medicinal applications [5]. Currently, GV has been used in the development of vaccines [7], magnetic resonance imaging (MRI) [8], ultrasound imaging [9], disease treatment [10], etc. (Fig. 1) For example, recent studies have used the stability and nanoscale characteristics of GV to deliver drugs [11, 12]. Whereas, some properties and structures of GV and the function of the protein encoded by the Gvp gene clusters have not been fully investigated. Due to the lack of information about the GVs, their utilization should need more study or research.

With the in-depth study of GV, an increasing number of individuals are focusing on the application of this technology. In this review, we highlight the recent advances in GV application, provide a brief overview of GV composition, physical and chemical properties, primary sources and regulatory proteins, and list the potential and obstacles of GV application.

## The source of GV

GVs were first discovered in cyanobacteria, thought to be “bubbles”, and have since been found in many other microorganisms. (Table 1) In 1965, Bowen and Jensen found that the “bubbles” in cyanobacteria were aggregated by cylindrical vesicles. These vesicles present a closed environment and have only a single wall 2 nm thick. After further study, Bowen and Jensen named this form of “fish bladder” vesicles GV [3].

**Table 1:** The different sources of GV

Microorganism		Reference
Archaea	*Halobacterium salinarum* PHH1	[13]
	*Halobacterium salinarum* PHH4	[14]
	*Halobacterium salinarum* NRC-1	[15]
	*Haloferax mediterranei*	[16]
	*Haloquadratum walsbyi*	[17]
Bacteria	*Anabaena flos-aquae*	[18]
	*Microcystis aeruginosa*	[19]
	*Planktothrix rubescens*	[8]
	*Serratia* sp. ATCC 39006	[20]
	*Polaromonas vacuolata* KCTC 22033^T^	[21]

PSB were the first microbes discovered to produce GV. They are the general name for bacteria that perform non-oxygen photosynthesis under anaerobic conditions. In addition to PSB, the other subset of bacteria can also produce GV. Heterotrophic bacteria are one of these. The majority of GVs produced by these bacteria are largely cylindrical, although even within the same bacterium, some GVs vary in size and shape [22]. Bacteria that produce GVs in heterotrophic bacteria were first discovered in extremely cold environments, such as the sulfate-reducing bacteria *Desulforhopalus vacolatus*, the psychrophilic bacteria *Psychromonas ingrahamii* and *Rhodoferax antarcticus* [23–26]*.* In addition, researchers have also found the presence of GV in *Serratia* sp. ATCC 39006 and *Polaromonas vacuolata* KCTC 22033^T^ [20, 21].

Another typical microorganism that produces GVs is halophilic archaea that live in a high-salt environment. Later research found that halophilic archaea such as *Halobacterium salinarum*, *Haloferax mediterranei*, *Heterocypris salina*, and *Haloquadratum walsbyi* contain up to 70 spindle-shaped or cylindrical GVs in their bodies [27]. Over the next few years, with the efforts of other researchers, the major characteristics and functions of GV were gradually discovered [6].

## The function of GV

Aharon Oren showed that the GVs produced by different microorganisms have different functions [6]. The GVs produced by PSB give them the buoyancy to move upward so that they can get enough light for photosynthesis to produce nutrients [5, 28]. After photosynthesis, the carbohydrate produced is much greater than the density of water, which can cause it to sink vertically. Thus, the mutual regulation of the buoyancy of GV with the density of carbohydrates allows microorganisms to float or sink to maintain nutrient balance depending on the environment [29]. GVs produced by halophilic archaea have a similar effect. Because the solubility of oxygen in saturated salt solutions is very low and halophilic archaea grow in high-salt environments, they are susceptible to oxygen starvation. GVs can lift these halophilic archaea from the bottom of low-oxygen waters to an appropriate oxygen concentration level by increasing upward buoyancy [30]. In addition, there are several unique halophilic archaea, for example, *Haloquadratum walsbyi*, which is so thin that GV divides them into two cytoplasms [17, 31, 32]. Some special bacteria, for example, *Orenia sivashensis*, whose GV is located between the inner membrane of the exospore and the spore layer, may be related to spore movement [33].

## The structure and physical properties of GV

GVs are the largest protein-based assemblies ever reported, and their structures and properties are complex [34–37]. The study found that *Anabaena flos-aquae, Calothrix* sp. PCC 7601 and *Microcystis* sp. BC 84/1 could use the buoyancy generated by the GVs in their bodies and the density of the organic matter produced by their photosynthesis (the density of the organic matter is greater than that of water) to regulate their vertical movement [38, 39]. As mentioned above, the GV shape is very similar to the fish bladder, which can also be called spindle-shaped. Gas can freely penetrate the GV shell so that the gas inside and outside the vesicle can reach a balanced state [40]. The structure of GV produced by microorganisms is very stable due to their own needs for the environment. However, when GVs are under pressure, there is a critical point at which the GV irreversibly collapses [39]. The critical collapse pressure of GV in different microorganisms ranges from 0.09 MPa to 1 MPa. In deeper waters, microorganisms tend to form narrower GVs due to the regulation of the Gvp gene [41–43]. These GVs have a shell that allows the gas to pass freely through the surrounding environment, achieving both internal and external balance. For the currently studied GV structure, it is generally believed that it is mainly composed of GvpA to form a transverse “rib”-shaped shell, and then the outer surface is reinforced by GvpC [37, 44].

Recently, Huber et al. described the molecular structure of GV in atomic detail in their report [45]. They collected and purified the GV produced by engineered *E. coli* and observed its structures by cryo-electron microscopy. The results showed that the shell of the GV was only one or two peptide layers thick. Further analysis of the composition of the GV shell showed that it was reinforced by hydrogen bonds and salt bridges [45]. At the same time, the inner shell of the GV is hydrophobic, and when the gas passes through the channel in the GV shell, it is found that the pores in the GV shell are large, and these pores are caused by α1 helix of the GvpA, which can allow the external gas to diffuse freely into the vesicle. Their study also found that GvpA can self-assemble into two helical half-shells and close at the cone apex, after which the two half-shells are joined by the characteristic arrangement of GvpA monomers to form the GV structure of a hollow helical cylinder. In a previous study, Dutka et al. using cryo-electron tomography also found that the GV shell is formed by helical filaments of the highly conserved GvpA subunit and that GvpC is attached around the GvpA shell to form a helical cage that reinforces the overall structure [46]. Huber’s experiments showed the GvpC in GVs can tightly bind to the α2 spiral in the GV, which also confirmed that GvpC is a protein that can strengthen the outer structure of GVs [45]. In addition to studying the structure of GV, researchers also explored the molecular mechanism of GV production.

## Associated proteins of GV

The reason why these microorganisms can produce GVs is their ability to express specific GV proteins (Table 2). The most important of these is the structural protein GvpA, which is a small hydrophobic protein that forms the hollow protein structure of GV [50]. GvpA constitutes GV in various bacteria and archaea, such as *Haloarchaea* sp. [30], *Mediterranean* sp. [51], *Anabaena* sp. [52], and others. GvpC is another coat protein. It is the second structural protein involved in the formation of GV. GvpC is a different peptide from GvpA, which has good hydrophilicity. After the study, it was found that GvpC has the function of strengthening the GV shell [5].

**Table 2:** Proteins associated with the formation of GV from different sources

Species	Associated proteins	Reference
*Halobacterium salinarum* PHH1	GvpA, GvpC, GvpN, GvpD, GvpE, GvpF, GvpG, GvpH, GvpI, GvpJ, GvpK, GvpL, GvpM	[13]
*Microcystis aeruginosa* PCC7806	GvpA1, GvpC, GvpF, GvpG, GvpJ, GvpK, GvpN, GvpV, GvpW, GvpX	[47]
*Polaromonas vacuolata* KCTC 22033^T^	GvpA1, GvpA2, GvpA3, GvpC, GvpF1, GvpF2, GvpF3, GvpG, GvpH, GvpN, GvpV, GvpW, GvpX, GvpZ	[21]
*Bacillus megaterium* VT1660	GvpA, GvpB, GvpF, GvpG, GvpJ, GvpK, GvpL, GvpN, GvpP, GvpQ, GvpR, GvpS, GvpT, GvpU	[48]
*Streptomyces* sp. CB03234-S	GvpA, GvpF, GvpG, GvpJ, GvpK, GvpL, GvpO, GvpS	[49]

In addition to GvpA and GvpC, many other GV proteins have been found to be involved in GV synthesis, either regulating the expression of core proteins or assisting in GV shell synthesis [53]. Several GV proteins are involved in the process of GV synthesis: GvpE is responsible for activating transcription, and GvpD has an inhibitory effect on GV formation. GvpE can activate transcription and thus promote the expression of core proteins, whereas GvpD deficiency promotes GvpE cleavage and thus inhibits GV synthesis [16, 39]. In addition, some important accessory GV proteins are closely related to GV synthesis. For example, GvpJ and GvpM are essential hydrophobic accessory GV proteins with sequences and functions similar to GvpA [54]. GvpF is an important accessory protein because it is the only protein that binds to GvpA. GvpL is homologous to GvpF but has the opposite function, binding to all GV proteins except GvpA [55, 56]. In addition, there is another energy-providing GV protein, GvpN, which can hydrolyze adenosine triphosphate (ATP) to release energy due to its nucleoside triphosphate (NTP) binding/AAA+ domain [20]. GvpV, W, X, Y, and Z are specific types of proteins, such as GvpV, which is related to the growth and maturation of functional GVs; GvpW, which is a putative homolog of GvpL; and GvpX, which is somewhat similar to GvpJ [20, 47, 53]. In short, there are many proteins related to GV synthesis, and the mechanism of influence of some GV proteins is not been fully understood, which needs to be further studied and applied.

## Application progress of GV

The appearance of GVs has aroused great interest in the fields of biomaterials and biomedicine and has become one of the current research hotspots. Based on their physical and structural properties, they are used as stable contrast agents, reporter genes, or biosensors for a variety of imaging modalities such as ultrasound [9], MRI [57], optical imaging [58], etc. Based on their biochemical properties, they are being developed for use in research delivery vectors, novel specific vaccines and disease treatment. (Table 3) GV is being used in all directions; recent studies have integrated gene fragments producing GV into mammalian cells for expression, achieving effective gene and cell therapy tracking in vivo [9]. At the same time, GV can also be genetically and chemically modified, and based on this, many derived biomedical materials have been developed for the prevention, diagnosis, and treatment of diseases [60, 61].

**Table 3:** Different application strategies and related mechanisms of GV

Abbreviations		Abbreviations	
Ultrasound imaging	Contrast agent	Gas vesicles filled with gas can enhance the contrast of ultrasound signals between tissues	[59]
	Reporter gene expression product	The ARG was transferred into the cells to generate gas vesicles in the cells, which enabled cell visualization	[9]
	Ultrasonic biosensor	Engineering GvpC to detect enzyme activity	[60]
Hyperpolarized xenon MRI	Contrast agent	Hyperpolarized 129Xe can exchange with the interior of the GV	[61]
Optical coherence tomography (OCT)	Contrast agent	The air in the GV results in a strong scattering of incident light, improving the contrast of OCT signals	[58]
Material delivery	Delivery vehicles	Modify GvpA or GvpC to bind target proteins or genes	[62]
Vaccine	Antigen presentation	Engineering modification GV presenting antigens to stimulate an immune response	[63]
Others	Coagulant	GV collapse destroys cells and leads to the accumulation of proteins in extracellular organisms that promote clotting	[19]
	Open the vascular barrier	Ultrasound mediates GV to produce a cavitation effect to open blood vessels	[64]

### Ultrasound imaging

**Fig. 2:**
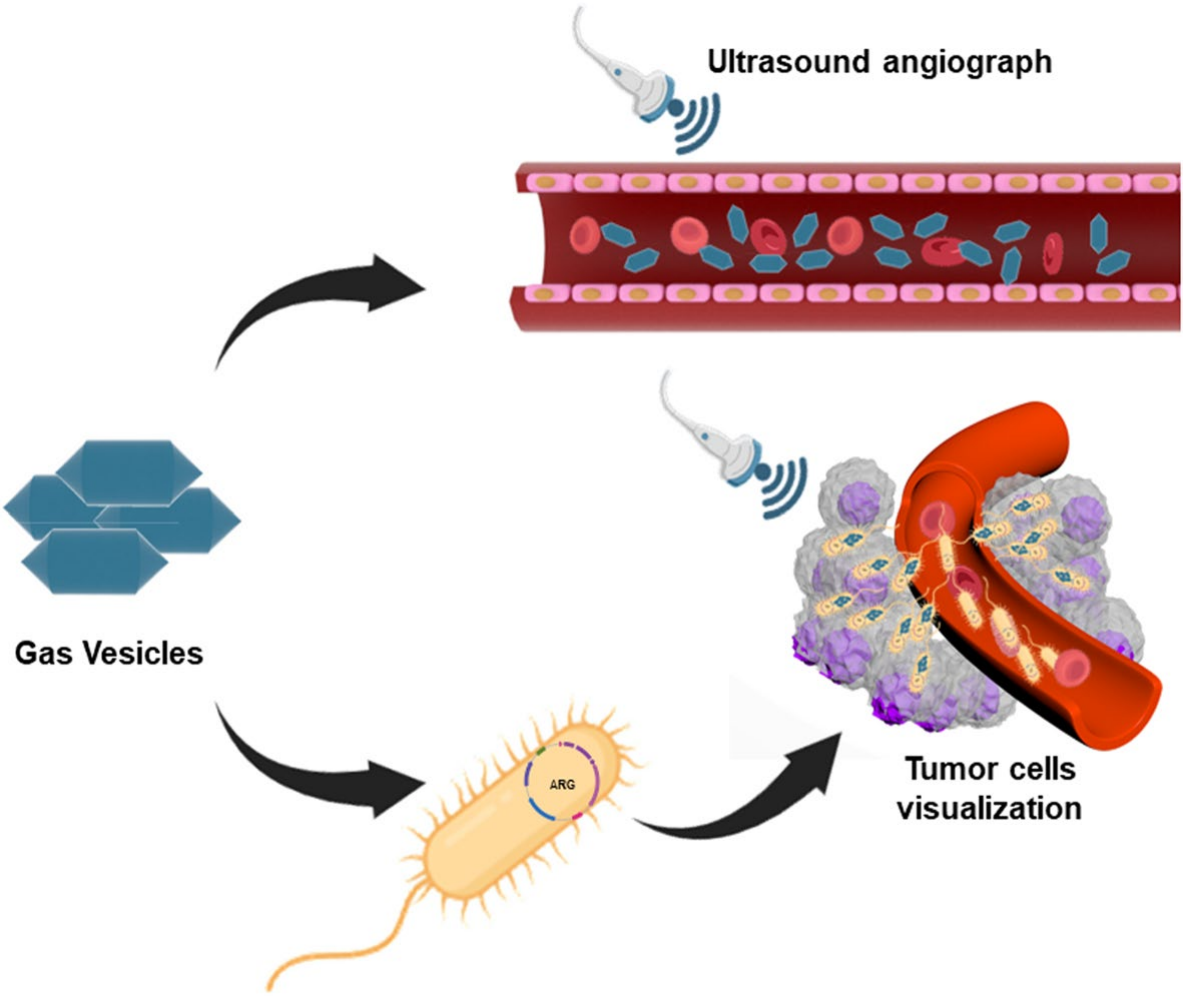
GV is used for ultrasound imaging. GVs can be injected into blood vessels as contrast agents to achieve ultrasound angiography. The genes expressing GV in bacteria can be delivered to tumor cells and then achieve ultrasound visualization of tumor cells

Microbubbles are the most common ultrasound contrast agent. In clinical diagnostics, microbubble-assisted ultrasound imaging is mainly used in the vascular system. In addition, a large number of preclinical studies have shown that microbubbles functionalized with specific ligands could enable molecular imaging of tissues and organs such as tumors or blood vessels by binding to molecular targets [65]. However, the size of the microbubbles limits their extravasation in the vascular lumen, and the short circulation time in the vasculature limits their application in imaging [66]. The study by Lakshmanan et al. showed that GVs produced by cyanobacteria and halophilic archaea could scatter sound waves and produce significant ultrasound contrast at sub-nanomolar concentrations [61]. In addition, these microbial-derived GVs, due to their smaller size and better stability, can be used as new ultrasound contrast agents to overcome the shortcomings of existing ultrasound contrast agents and realize ultrasound imaging at the biomolecular or cellular level. (Fig. 2) Ling et al. injected GVs into blood vessels to evaluate the imaging effects of GVs in ultrasound angiography [59]. Further studies have shown that GVs injected into the body could attach to the surface of red blood cells and lead to hemodynamic enhancement, thus enabling effective ultrasound functional imaging. However, due to the surface properties of GV, it can adsorb apolipoproteins and immunoglobulins to form a “protein corona”, which shortens the cycle time in the body and affects the imaging results [59]. Subsequently, methoxypolyethylene glycols (mPEGs) were used to passivate the surface of the GV, which could reduce protein adsorption. Furthermore, by passivating the surface of the GV, its time in circulation is prolonged, allowing long-term ultrasound imaging observations [67]. In addition to ultrasound imaging of the vasculature, the team used it for ultrasound molecular imaging at the cellular level.

A reporter gene is a gene whose expression products can be detected. At present, a variety of reporter genes have been developed. For example, optical reporter genes can achieve optical imaging of tissues by expressing substances with bioluminescence or fluorescence ability [68]. Based on the ultrasound imaging characteristics of GV, the gene producing GV has been applied to the research and development of the acoustic reporter gene (ARG) to achieve fine ultrasound imaging of deep tissues. Lakshmanan et al. introduced 2 GV-producing gene clusters as acoustic reporter genes into bacteria and mammalian cells, respectively. The authors found that the above two gene clusters could be expressed in bacteria and mammalian cells to produce GVs, which significantly improved the acoustic contrast. Expression of ARG in bacteria enabled non-invasive ultrasound imaging of bacteria embedded in tumors, while expression of ARG in mammalian cells enabled long-term monitoring of cancer cells [9]. The development of this strategy will be conducive to the wide application of GV.

Another application of GV is as an ultrasonic biosensor for the detection of cell enzyme activity. Lakshmanan et al. have shown that the variant of GvpC can be engineered to generate protease-activated GV, in which the variant of GvpC was incorporated into an amino acid sequence that could be recognized and acted upon by a specific protease [60]. Constitutively, active tobacco etch virus (TEV) endopeptidase, calcium-dependent mammalian protease calpain, and persistent bacterial protease ClpXP-responsive GV have been developed to reveal the activity of proteases under ultrasound radiation and to demonstrate the ability of biosensors to image in vitro, live bacteria and in vivo. In summary, GV is being used as an ultrasound contrast agent and acoustic biosensor due to its ability to be modified by surface GvpC modification and chemical functionalization. With further research, Shapiro et al. proved that GV produced by different species responds to different sound pressures [69]. This property can be exploited to realize ultrasound multiple imaging based on different types of GVs. Some similar experiments have shown that engineered GVs respond to sound pressure by nonlinear mechanical deformation, which allows them to be selectively imaged by tailored amplitude modulation strategies [70]. In addition, Yang’s study also showed that as the concentration of GV increases, the ultrasound signal generated by it was also gradually enhanced [71], and with the further development of the biomedical field, GV will expand into more application scenarios.

### MRI

In addition to its use as an ultrasound contrast agent, GV can also be used as a contrast agent for MRI. Chemical exchange saturated transfer (CEST) MRI is an emerging in vivo molecular imaging technique based on proton exchange between saturated protons and the surrounding medium with high sensitivity [72]. In a subsequent study, Lakshmanan et al. discovered that the atoms of the hyperpolarized ^129^Xe dissolved in the water medium could exchange with the interior of the GV through a special chemical shift, enabling the contrast of the MRI signal to be amplified using CEST pulse sequences and obtaining high-quality MRI images [61]. The results showed that GV could improve the image contrast of hyperpolarized xenon MRI and increase the molecular sensitivity by 10^5^ times through non-equilibrium spin polarization. Using the HyperCEST technique, MRI can detect GVs at a picomolar concentration. It has promoted the development of hyperpolarized gas MRI technology.

Recently, based on previous studies, Ryota Mizushima et al. extracted GV-expressing gene clusters from *Planktothrix rubescens/agardhii* and reprogrammed them into human breast cancer KRL-4 cells [8]. The results showed that GV-like particles (GVLPs) could be expressed in the engineered cells, and the MRI signal of hyperpolarized xenon was also detected by the device, indicating that it could be used as a HyperCEST MRI contrast agent. The study achieved the expression of GV in mammalian cells and proved that it has the same function as ordinary GV and could enhance the contrast of HyperCEST MRI, which provides a possibility for the application of GV in the human body.

Previous studies have found that in MRI, air can produce signals of different intensities compared with diamagnetic water, which can be observed in lung MRI images [73]. Lu et al. deduced and confirmed that air-filled GV also had this effect [57]. They found that a nanoscale magnetic field gradient was generated around a single GV in water, and this magnetic field could promote the T2/T2* relaxation of water molecules, resulting in a decrease in signal strength of the T2 and T2*-weighted images [74], producing a negative enhancement effect. Then, GVs were ruptured by ultrasound, MRI images were taken before and after the destruction of the GVs, and MRI signals of the GVs were obtained using the MRI subtraction technique. Finally, they performed MRI of the striatum of the mouse brain using GV. The results showed that GV combined with ultrasound could significantly enhance the contrast of MRI.

### Optical imaging

Early studies have found that microbubbles can be used as contrast agents to enhance OCT imaging [75]. GV has similar structural characteristics to microbubbles and has the potential to be used as a contrast agent for OCT imaging. Lu et al. studied OCT images of hydrogels of three different GV types: *Anabaena flosa-quae*, *Halobacterium NRC-1*, and *Bacillus megaterium*. The authors found that the air content of GVs caused these nanostructures to strongly scatter incident light, making GV-based contrast agents or GVs-expressing cells visible on OCT images [58]. All three types of GVs had obvious OCT contrast images, among which the strongest scattering intensity was the GV produced by *Halobacteria NRC-1*. Recently, Schrunk et al. used the bioorthogonal label FlAsH to achieve detailed fluorescence visual detection of intracellular GV [76]. They used GvpA to carry the fluorescent label six-amino acid tetracysteine (TC) tag and then prepared GV. This study further demonstrated that GV could be successfully fluorescently visualized in HEK 293 T cells, which can be applied to study the size and distribution of GV in cells. Because the genes encoding GVs could be integrated into specific microorganisms so that they could be expressed in the microorganisms, they have been widely used in recent years for studies of microscopic imaging and monitoring in organisms.

### Delivery vector

**Fig. 3:**
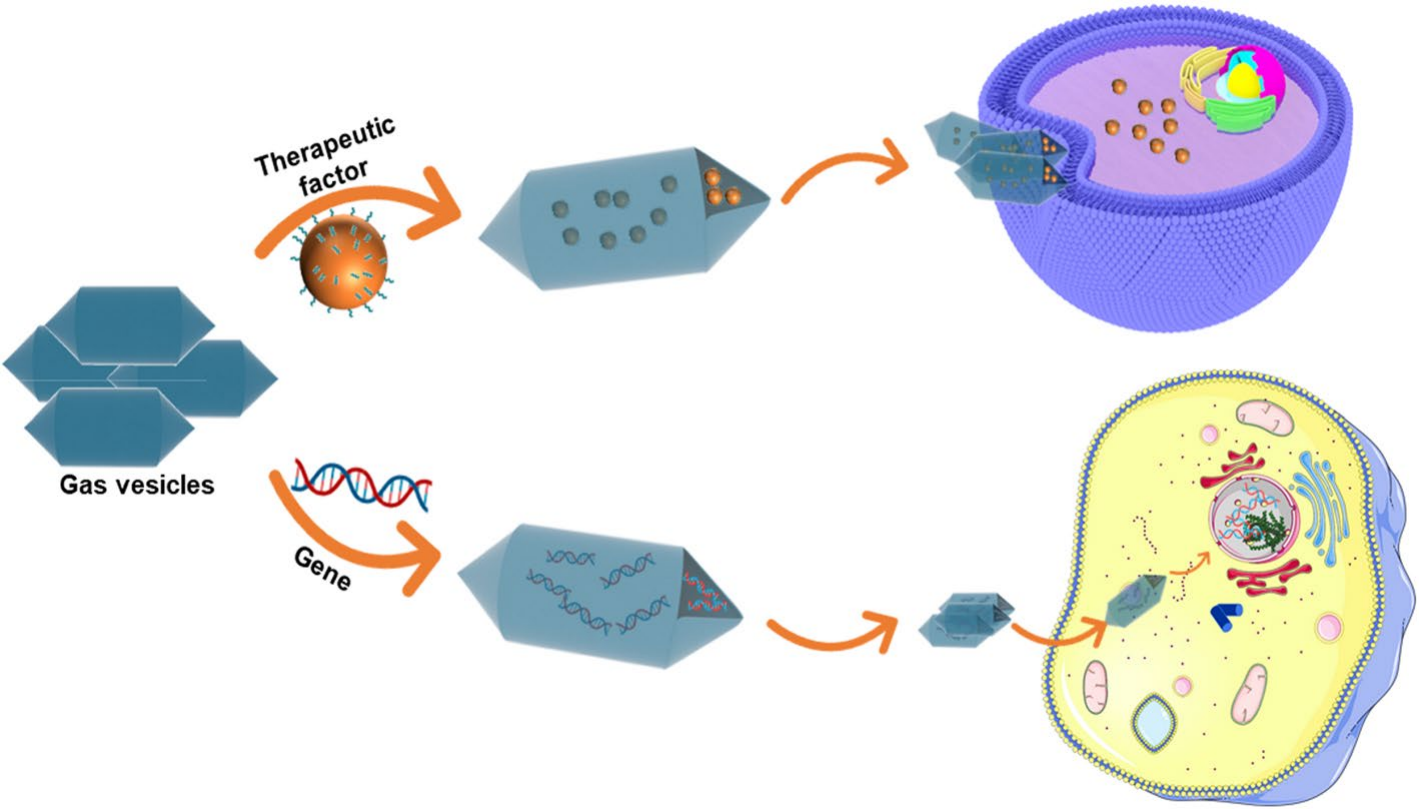
GV is applied to delivery. GV delivers therapeutic factors and genes at the cellular level

In addition to its imaging applications, GV is a highly efficient carrier. (Fig. 3) Due to the high cost and insufficient oxygen affinity of the conventional oxygen carrier hemoglobin, its widespread application is limited. Based on the physical and chemical properties of GV and its ability to exchange gases, Anand Sundararajan et al. developed a strategy to use GV to deliver oxygen [18]. They prepared oxygen-carrying GV, and the results showed that the glucose utilization rate (GUR) of the cells increased significantly by 30% in the 1.8% oxygen-carrying GV medium, indicating that GV can be used as an oxygen carrier. With the in-depth study of GV, researchers found that GV surface proteins can be modified to achieve the transport and delivery of proteins, nucleic acids, and other substances. To treat endotoxemia, Arjun Balakrishnan fused mouse bactericidal/permeability-increasing protein (BPI) with GvpC on the surface of GV nanoparticles (GVNPs, the GVs are produced by *Halobacterium* sp. NRC-1) to produce mBPIN-GVNPs with the effect of treating endotoxemia [77]. Through in vivo transfer, mBPIN-GVNPs showed a significant protective effect, which not only prevented the occurrence of endotoxic shock in mice but also inhibited various inflammatory responses caused by lipopolysaccharide and significantly improved the survival rate of mice with endotoxemia. With the further study of GV and the development of genetic engineering technology, the application of GV as a carrier has made new progress. Ram Karan et al. selectively modified the cysteine residues of the GvpA on the GV surface, then selectively labeled the GV-modified GvpA through a cysteine-maleimide-mediated reaction and coupled horseradish peroxidase (HRP) to the GV surface through the streptavidin-biotin effect. To further simplify the coupling process, Ram Karan implemented protein coupling using the SpyTag003- SpyCatcher003 system [62].

With the development of biological technology, the application of GV has been further developed. Recently, Xie et al. used GV as a cavitation nucleus to carry the E-cadherin nuclear gene [10]. After GV entered the cells, the ultrasound-induced gene-carrying GVs produced a cavitation effect in cells and then delivered plasmid DNA directly to the nucleus to realize gene transcription and expression, effectively improving the transfection efficiency of foreign genes. Due to the precise targeting characteristics of ultrasound and the good biocompatibility and stability of GV, spatiotemporal-controlled gene delivery in the cell nucleus is possible.

### Vaccine development

In addition to being a delivery vehicle, GVNPs can also be used to regulate the body’s immune response. Sremacet and Stuart integrated simian immunodeficiency virus (SIV)-related gene fragments (Tat, Rev., and Nef1) into GVNP surfaces to make specific vaccines [78]. The recombinant GV (r-GV) induced an immune response similar to the long-term immune response induced by viral protein and also increased the expression levels of IL10 and other factors in vivo. Among them, the Tat-GVNP vaccine induced the strongest immune response. In addition, another research team integrated SopB (a secreted inosine phosphate effector protein) fragments with GVNP to stimulate the body’s immunity against *S. enterica serovar Typhimurium*, and studies showed that SopB-GVNP provided the body with a stronger and more durable immune response while reducing bacterial load [79]. Studies such as the above have found that GVNPs have advantages over live attenuated vaccines and that bioengineered GVNPs can act as an adjuvant and delivery system to induce immune responses by presenting antigens to antigen-presenting cells (APCs) [7].

In addition, GV can also act as an immune enhancer to increase immunogenicity and avoid side effects. Childs et al. engineered GV into r-GV and then integrated the gene fragment expressing the chlamydia antigen protein into r-GV [63]. It was found that the modified r-GV could successfully express the immunogenic chlamydia antigen recognized by animal and human serum while increasing the level of inflammatory factors. Their study showed that r-GV has good stability in human and animal cell lines, and at the same time, it also has time-dependent self-degradation properties, allowing for slow, time-controlled release of substances [63]. These results showed that GV could be engineered to express different pathogen peptides to stimulate immune responses and exhibit immune memory even in the absence of immune adjuvants [80, 81], which could also enhance immunogenicity without side effects [82], providing a new framework for vaccine development and aiding in the research and development of new therapeutic strategies.

### Other applications

In addition to the above, GVs can be used in other scenarios. Li et al. applied high-frequency ultrasound to the GVs produced by *Microcystis aeruginosa*, resulting in the collapse of the GVs [19]. Cavitation of the GVs would produce mechanical and chemical effects, that further promote the release of cell contents and even lead to cell lysis [83]. Finally, it leaded to increased protein accumulation in the extracellular organic matter. Previous studies have found that a small amount of proteins accumulation could promote blood clotting, while a large amount of protein accumulation could improve blood clotting [84–86]. By adjusting the ultrasound parameters, it can be controlled whether to promote coagulation. Similarly, to address the low delivery rate and carrier safety issues faced by cardiac microRNA therapy, Wang et al. induced GV cavitation using low-intensity pulsed ultrasound to open the vascular barrier [87], and deliver antagomir-155 to the allograft heart [64]. The small size of GVs allowed them to produce cavitation effects while minimizing potential damage. It provides an idea for the development of ultrasound cavitation-assisted, targeted, and powerful microRNA therapy strategies.

## Discussion

Recent studies have proved that GV has the advantages of simple structure, small size, strong stability, and high biocompatibility, and it has been widely used in a variety of single-mode imaging, protein delivery, antigen presentation, and other fields, providing a new way for disease prevention, diagnosis, and treatment. With further development, the limitations of GV are gradually revealed. In terms of imaging, compared with traditional ultrasound contrast agent microbubbles, GV has a smaller volume and lower echo intensity. Gas vesicles are also extremely selective in their application as biosensors for specific assays such as enzyme activity. To avoid non-specific clustering, different molecular designs of GV are required according to specific conditions, which limits the wide application of GV.

Studies have found that, compared with traditional subunit vaccines, GV can produce a stronger immune response while being more biocompatible. In recent years, a variety of new vaccines have been developed based on GV, such as the *Salmonella enteric serotype typhimurium* vaccine, the *Chlamydia* vaccine, etc. [63, 82]. However, GV also has some limitations in the development of new vaccines. Unlike other antigen-presenting systems, GV can only present antigens on the surface in terms of delivery strategy. At present, the surface of GV can only present antigen protein fragments, not related plasmid DNA or mRNA. In terms of antigen protein delivery, GV is suitable for the delivery of bacterial antigen proteins and some viral, eukaryotic, and cancer antigens. For most of the antigen proteins that require modification after expression translation, the GV delivery system makes it difficult to achieve ideal results.

In short, the application development of GV faces many challenges, but it still has great development potential. In imaging, novel universal contrast agents corresponding to different modalities can be developed by studying the vesicle shell structure or composition of different GVs. For vaccine development, a team of researchers recently achieved delivery of the E-cadherin nuclear gene into cells via GV [10], and then the system can be improved to achieve the delivery and expression of antigen-related gene fragments. In addition, the gene expressing GV has been expressed in *E. coli* [9], and in the future, the GV antigen-presenting system can be combined with probiotic-related biological technologies to develop new vaccines that can be taken orally. With the joint development of multi-field technologies, the limitations of GV will be greatly reduced and applied to more scenarios.

## Data Availability

No datasets were generated or analysed during the current study.

## Abbreviations

APCs: Antigen-presenting cells
ARG: Acoustic reporter gene
ATP: Adenosine triphosphate
BPI: Bactericidal/permeability-increasing protein
CEST: Chemical exchange saturated transfer
DNA: Deoxyribonucleic acid
GUR: Glucose utilization rate
GV: Gas vesicle
GVLPs: GV-like particles
GVNP: Gas vesicle nanoparticles
GvpA: Gas vesicle protein A
HRP: Horseradish peroxidase
mPEGs: Methoxypolyethylene glycols
MRI: Magnetic resonance imaging
NTP: Nucleoside triphosphate
OCT: Optical coherence tomography
PSB: Photosynthetic bacteria
r-GV: Recombinant gas vesicle
SIV: Simian immunodeficiency virus
TC: Tetracysteine
TEV: Tobacco etch virus
